# Prophylaktische Fasziotomie bei Tibia-Osteotomien: funktionelle Ergebnisse

**DOI:** 10.1007/s00113-021-01070-y

**Published:** 2021-08-25

**Authors:** Nikolaus Degen, Tobias Randeu, Florian Wolf, Julian Fürmetz, Ekkehard Euler, Wolfgang Böcker, Peter Helmut Thaller

**Affiliations:** 1 3D-Chirurgie, Klinik für Orthopädie und Unfallchirurgie, Muskuloskelettales Universitätszentrum München (MUM), Klinikum der Universität München, LMU München, Campus Innenstadt, Ziemssenstr. 1, 80336 München, Deutschland; 2 Klinik für Orthopädie und Unfallchirurgie, Muskuloskelettales Universitätszentrum München (MUM), Klinikum der Universität München, LMU München, Campus Innenstadt, Ziemssenstr. 1, 80336 München, Deutschland

**Keywords:** Beinlängendifferenz, Marknagel, Bewegungsausmaß, Kraft, Kompartmentsyndrom, Leg length discrepancy, Intramedullary nailing, Range of motion, Strength, Compartment syndrome

## Abstract

**Hintergrund:**

Bei Tibia-Osteotomien (TO) mit Marknagelfixierung kann eine minimal-invasive, prophylaktische Fasziotomie (PF) der Extensorenloge zur Prävention eines postoperativen akuten Kompartmentsyndroms (KS) erfolgen. Bislang sind keine Studien über die Effekte von TO oder PF auf spezifische Funktionen der Extensoren bekannt.

**Ziel der Arbeit:**

Die Untersuchung der Funktion nach PF und TO an Patient*innen ohne präoperative funktionelle Einschränkung.

**Material und Methoden:**

In 41 Fällen (28 Frauen, 13 Männer) erfolgte durchschnittlich 6,1 Jahre nach elektiver TO mit PF und Marknagelfixierung eine Befragung zur klinischen Funktion. In 23 Fällen wurden die isometrische Kraft und der „range of motion“ (ROM) der Dorsalextension (DE) des oberen Sprunggelenks (OSG) gemessen. Die Kraft wurde als Test auf klinische Relevanz mit der 10 %-Normperzentile verglichen, sowie zur Gegenseite.

**Ergebnisse:**

In durchschnittlich 86 % der Fälle wurde von keinen oder geringen funktionellen Einschränkungen der Extensoren berichtetet. Die mittlere Kraft zeigte keine signifikante Abweichung von der geschlechterspezifischen 10 %-Normperzentile, aber war im Seitenvergleich auf der operierten Seite mit 16,0 ± 6,5 kgf signifikant geringer als auf der Gegenseite mit 17,5 ± 6,3 kgf (*p* < 0,01). Die subjektive Einschränkung der DE im OSG korrelierte deutlich negativ mit der ROM (r_s_ = −0,46, *p* < 0,05).

**Diskussion:**

Die Ergebnisse lassen auf eine geringe Häufigkeit subjektiv relevanter funktioneller Einschränkungen der Extensoren schließen. Die vorgefundene Kraftminderung war nicht klinisch relevant. Subjektive Einschränkungen scheinen vorrangig durch Abnahme des ROM verursacht zu sein.

## Abstract (en)

**Background:**

Intramedullary nailing in tibial osteotomy (TO) can be combined with minimally invasive prophylactic fasciotomy (PF) of the anterior compartment of the lower leg to prevent postoperative acute compartment syndrome (CS). So far no studies are available on the effects of TO or PF on specific functions of the extensor muscles.

**Objective:**

To investigate the medium-term outcome after PF in TO in patients without preoperative functional impairment.

**Material and methods:**

In 41 cases (28 women, 13 men) the patients were questioned with respect to clinical function on average 6.1 years after elective TO and PF fixed with intramedullary nails. Of the cases 23 were examined measuring isometric strength and range of motion (ROM) of dorsiflexion (DF) of the foot. Strength was compared to the 10% standard percentile to test for clinical relevance and to the contralateral side if applicable.

**Results:**

In an average of 86% of the cases no or minor functional impairment of the extensors was reported. The DF of the foot was rated as the leading impairment. Mean strength did not differ significantly from the gender-specific 10% standard percentiles but was significantly lower on the operated side with 16.0 ± 6.5 kgf compared to the healthy side with 17.5 ± 6.3 kgf (*p* < 0.01). Subjective impairments of DF of the foot correlated clearly negatively with ROM (r_s_ = −0.46, *p* < 0.05) but not with strength.

**Conclusion:**

The results indicate a low occurrence of subjectively relevant functional impairment of the extensor muscles. The decrease of strength was not found to be clinically relevant. Subjective impairments appeared to be caused by a decrease of ROM, not strength.

Tibia-Osteotomien (TO) zur Achsen‑, Längen- und/oder Torsionskorrektur gehen mit einem erhöhten Risiko für ein akutes Kompartmentsyndrom (KS) einher. Am häufigsten ist die Extensorenloge betroffen, welche besonders bei minimal-invasiven Bohrlochosteotomien ebenso minimal-invasiv gespalten werden kann. Eventuell resultierende funktionelle Einschränkungen der Extensorenfunktion wurden bislang nicht untersucht.

Neben dem therapeutischen Stellenwert der Fasziotomie in der Behandlung des akuten und chronischen KS wird diese bei Eingriffen mit erhöhtem Risiko eines postoperativen akuten KS auch prophylaktisch durchgeführt. Hierzu zählen besonders die proximalen, diaphysären TO zur Achsen‑, Torsions- und/oder Längenkorrektur in Marknageltechnik. Sowohl die Einblutung aus dem eröffneten und oft aufgebohrten Markraum in das Kompartiment als auch eine Muskelschwellung infolge der intraoperativen Manipulation werden als Pathomechanismen diskutiert. Die möglichen Folgen einer unzureichenden Therapie beinhalten dauerhafte sensible und motorische Störungen der betroffenen Extremität, ischämische Nekrosen mit hierdurch entstehenden Kontrakturen, Verlust der Gliedmaßen und in seltenen Fällen den Tod [11].

In unserer Arbeitsgruppe trat zwischen 1999 und 2003 unter ca. 60 der genannten Operationen am Unterschenkel in 3 Fällen ein postoperatives akutes KS auf. In der Folge wurde die minimal-invasive prophylaktische Fasziotomie (PF) der Extensorenloge des Unterschenkels als routinemäßige Maßnahme für minimal-invasive Korrekturosteotomien an der Tibia eingeführt. Uns sind nur 2 weitere Studien mit Inzidenzangaben bekannt; diese beschreiben jeweils einen Fall von akutem KS nach TO und Marknagelung in einem Kollektiv von 12 bzw. 13 Fällen [1, 10]. Angesichts der physiologischen Funktion der Muskelfaszie und postoperativ wiederholt festzustellender Muskelhernien stellt sich die Frage nach den Auswirkungen der PF bei TO auf die subjektive und objektive Extensorenfunktion ([5]; Abb. 1).

**Figure 1:**
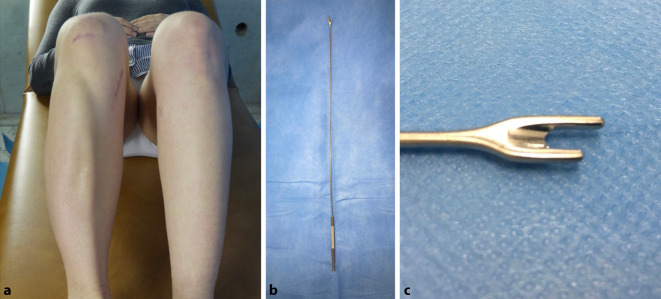

Bisherige Studien zum Bewegungsausmaß („range of motion“, ROM) nach TO fokussieren sich auf das Kniegelenk nach hohen TO oder auf das obere Sprunggelenk (OSG) nach distalen TO [15, 17]. Andere Arbeiten zur Beweglichkeit des OSG nach Marknagelung stammen aus der Frakturversorgung, was eine potenzielle Muskelschädigung bereits präoperativ impliziert [12, 14].

Es wurden jedoch keine Ergebnisse zu subjektiver Gelenkfunktion oder Messparametern wie Kraft und Mobilität gefunden. Diese sind nötig zur Abwägung des Nutzen-Risiko-Verhältnisses der Eingriffe durch Arzt und Patienten, insbesondere bei Patienten mit gering ausgeprägten Deformitäten.

Ziel der Studie war es daher, 1) die klinische Extensorenfunktion aus Patientensicht zu erheben und 2) die Parameter Kraft und ROM der DE im OSG als wichtigste klinische Funktionen zu messen an einem Patientenkollektiv ohne vorhergehende Beeinträchtigung mit mittlerem Nachuntersuchungsintervall. Zudem sollte 3) eine Subgruppenanalyse weitere Hinweise zur Pathogenese subjektiver Beeinträchtigung geben.

## Material und Methoden

### Operationstechnik und Patientenkollektiv

In allen Fällen erfolgte vor der minimal-invasiven Bohrlochosteotomie am proximalen Tibiaschaft über dieselbe, maximal 2 cm lange Hautinzision eine subkutane, komplette PF des anterioren Kompartiments nach proximal und distal. Hierfür kam ein spezielles, feines Fasziotom zur Anwendung (Abb. 1b, c und 2).

**Figure 2:**
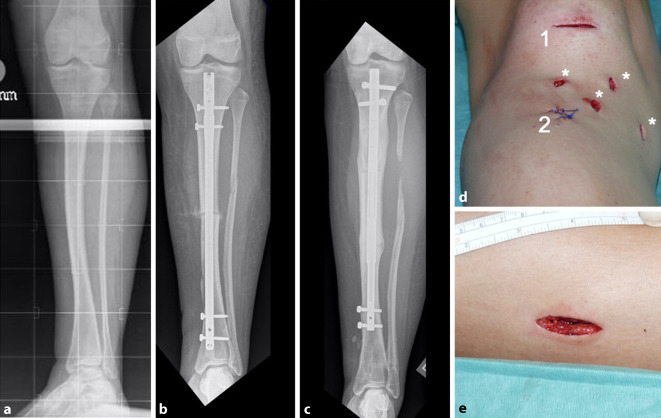

Achsen- und/oder Torsionskorrekturen wurden mittels Expert Tibia Nail® (Fa. DePuy Synthes) fixiert, welcher gelegentlich intraoperativ planungsgemäß oder auch zur Feinjustierung mittels Biegepresse konfektioniert wurde [22]. Als Verlängerungsmarknägel kamen in chronologischer Reihenfolge folgende Modelle zum Einsatz [21]: Fitbone® TAA (Fa. Wittenstein Intens, Igersheim, Deutschland), ISKD® (Fa. Orthofix, Verona, Italien), Phenix® (Fa. Phenix® medical, Paris, Frankreich) und Precice® (Fa.NuVasive, San Diego, CA, USA). Alle Eingriffe wurden von demselben Operateur durchgeführt (P.H.T.).

Eingeschlossen wurden 159 Fälle von Längen‑, Achsen- und/oder Torsionskorrekturen der Tibia mittels konventionellen Marknägeln oder vollimplantierbaren motorisierten Verlängerungsmarknägeln seit 2003. Ausgeschlossen wurden Fälle mit weniger als 3 Monate zurückliegendem Behandlungsabschluss (Implantatentfernung) sowie Fälle von muskuloskeletalen oder neurologischen Vorerkrankungen wie Poliomyelitis, Rachitis, M. Blount, neuropathischen Erkrankungen, Muskeldystrophie, Fibulahypoplasie oder -aplasie, posttraumatischem KS, präoperativen Seitendifferenzen der Kraftgrade nach Janda oder des ROM im OSG > 5° zur jeweiligen Gegenseite und bereits präoperativ vorliegender Muskelhernie [9].

Nach Ausschluss von 73 Fällen wurde in *n* = 41 Fällen (28 Frauen, 13 Männer) die subjektive Einschätzung der Beschwerden mittels Fragebogen erhoben; in den übrigen 45 Fällen war eine Kontaktaufnahme nicht möglich oder wurde die Teilnahme abgelehnt.

Aus diesem Kollektiv konnten 23 Fälle (15 Frauen, 8 Männer) für die klinische Nachuntersuchung von Kraft und ROM der DE im OSG gewonnen werden.

### Datenerhebung

Im Fragebogen verglichen die Patient*innen die subjektiven Parameter „aktuelle Beschwerden bei der Fußhebung/Heben der großen Zehe/ – der übrigen Zehen“ anhand einer numerischen Ratingskala (NRS, 1–5) mit dem präoperativen Status.

**Figure 3:**
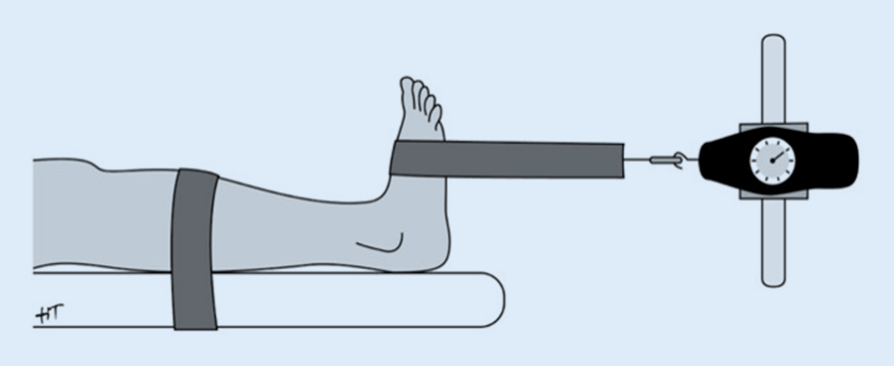

Die Kraft der DE wurde beidseitig als maximale isometrische Muskelkraft (MIMK) mittels eines vom Untersucher gehaltenen Zugkraftmessers („hand-held dynamometer“ – HHD; Gerät: Force Dial™ Model FDL Force Gage, Fa. Wagner Instruments, Greenwich, CT, USA) nach einem standardisierten Protokoll von Huber et al. bestimmt (Abb. 3; [8]). Die Einheit kgf („kilogramm force“, entsprechend der Kraft einer Masse von einem Kilogramm im Schwerkraftfeld der Erde) wurde aufgrund ihrer guten Nachvollziehbarkeit und direkten Vergleichbarkeit zu Normperzentilen beibehalten.

Die Messungen wurden unter Anwendung des „Make“-Tests durchgeführt, bei welchem die Patient*innen die Kraft über 2–3 s bis zum Maximum erhöht [2]. Für die Auswertung wurde der höhere von 2 erhobenen Kraftwerten verwendet. Isometrische Maximalkraftmessungen mittels HHD liefern mit stationären isokinetischen Kraftmessgeräten gut vergleichbare Resultate [19].

Die Erhebung des ROM erfolgte ebenso beidseits nach der Neutral-Null-Methode mittels Goniometer. Alle Nachuntersuchungen wurden von demselben Untersucher (T.R.) durchgeführt, um Intertester-Abweichungen zu verhindern [3, 4, 18].

### Auswertung

Die erhobenen Kraftwerte wurden mit Normperzentilen verglichen. Der einzige gefundene Anhaltspunkt für die Beurteilung der klinischen Relevanz einer Kraftminderung ist die von Huber et al. genannte Kraftminderung unter die 10 %-Normperzentile [8]. Demnach wurden für Frauen und Männer die Schwellenwerte von 13,0 kgf und 20,0 kgf definiert. Die auf diesen Werten und den Standardabweichungen nach Stoll et al. basierende Fallzahlberechnung ergab für die gemeinsame Signifikanzprüfung ohne Geschlechtertrennung gegen die 10 %-Normperzentile eine benötigte Fallzahl von 13 (einseitig; α = 0,05; Power: 80 %) [20].

Die Parameter MIMK und ROM der DE im OSG wurden jeweils mit der gesunden Gegenseite verglichen (nach Ausschluss von Fällen mit beidseitiger Operation: *n* = 11 Fälle; 7 Frauen, 4 Männer).

Zur Subgruppenanalyse wurden zunächst der NRS-Score in ein binäres Merkmal transferiert und ROM und MIMK zwischen Fällen mit geringer Beeinträchtigung (NRS1–2) und solchen mit höherer Beeinträchtigung (NRS 3–5) verglichen. Des Weiteren wurden ROM und Kraft (Differenz der geschlechterspezifischen 10 %-Normperzentile und der MIMK) bei Fällen mit Längenkorrektur gegenüber denen mit alleiniger Achsen‑/Torsionskorrektur betrachtet. Zuletzt wurden ROM, MIMK, und NRS zwischen Fällen mit und ohne Muskelhernie verglichen.

Die erhobenen Parameter wurden mittels Shapiro-Wilk-Test auf Normalverteilung geprüft und abhängig von ihrer Verteilung mittels einseitigem *t*-Test oder Wilcoxon-Test auf Unterschiede ihrer zentralen Tendenzen geprüft (Signifikanzniveau: *p* = 0,05).

## Ergebnisse

In 41 Fällen erfolgte eine Erhebung subjektiver Beeinträchtigungen mittels Fragebogen. Das durchschnittliche Alter zum Operationszeitpunkt betrug 24,1 ± 11,3 Jahre. Das mittlere Follow-up betrug 6,1 ± 4,1 Jahre. In 16 Fällen (12 Frauen, 4 Männer) wurde eine Achsen- oder Torsionskorrektur durchgeführt, in 25 Fällen (16 Frauen, 9 Männer) eine Längenkorrektur (4-mal ISKD®, einmal Phenix®, 7‑mal Fitbone TAA®, 11-mal Precice®, 2‑mal „lengthening over nail“). In 22 Fällen war die linke, in 19 Fällen die rechte Seite betroffen. Der Body-Mass-Index war mit 22,9 ± 3,9 kg/m^2^ im Normbereich.

**Figure 4:**
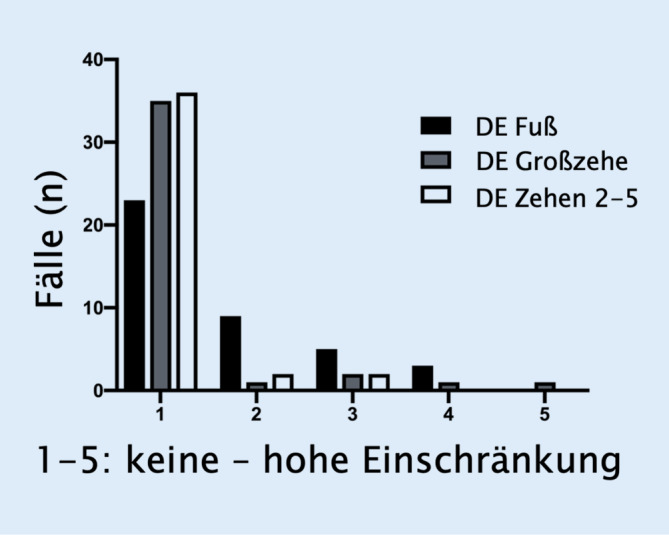

Durchschnittlich 86 % der Befragten gaben keine oder nur eine geringe Beeinträchtigung (NRS 1–2) der Extensoren an (78 %: Fußhebung, 88 %: Großzehenhebung, 93 %: Heben der übrigen Zehen; Abb. 4). Die DE im OSG zeigte mit einem mittleren NRS (1–5) von 1,7 ± 1,0 die größte Einschränkung, während für die DE der großen Zehe und übrigen Zehen noch geringere Beschwerden angegeben wurden (1,3 ± 0,9 bzw. 1,2 ± 0,7).

Die MIMK der DE im OSG (*n* = 23) lag bei 13,3 ± 3,5 kgf für Frauen und 18,6 ± 5,6 kgf für Männer. Der Vergleich mit den geschlechterspezifischen 10 %-Normperzentilen zur Prüfung der klinischen Relevanz zeigte keine signifikante Abweichung vom jeweiligen Testwert.

**Figure 5:**
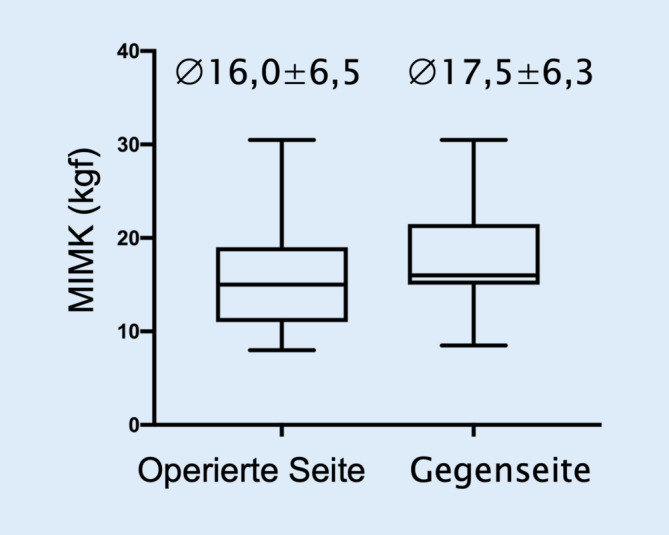

Im Seitenvergleich (*n* = 11) war die MIMK der DE im OSG auf der operierten Seite mit 16,0 ± 6,5 (11,6; 20,3) kgf im Mittel um 1,5 kgf geringer als auf der Gegenseite mit 17,5 ± 6,3 (13,3; 21,7) kgf, entsprechend einer statistisch signifikanten Kraftdifferenz von 8,6 % (*p* < 0,01; Abb. 5).

Zwischen dem ROM der DE im OSG auf der operierten Seite mit 14,4 ± 5,8 (10,5; 18,2) ° und auf der nichtoperierten Seite mit 16,0 ± 7,1 (11,3; 20,8) ° bestand kein signifikanter Unterschied.

Die Subgruppenanalyse ergab für Fälle mit keinen oder nur geringen subjektiven Beschwerden (NRS 1–2) einen signifikant größeren ROM mit 13,6 ± 7,0 (9,9; 17,3) ° als für Fälle mit stärkeren Beschwerden (NRS 3–5) mit 8,0 ± 2,3 (5,6; 10,4) ° (*p* < 0,05). Für die MIMK war zwischen diesen Gruppen hingegen kein signifikanter Unterschied festzustellen. Analog hierzu korrelierten subjektive Beschwerden signifikant negativ mit dem ROM, nicht aber mit der Kraft (Tab. 1).

**Table 1:** 

Beschwerden: Parameter	NRS 1–2	NRS 3–5	Signifikanz	Korrelation Beschwerden – ROM	Korrelation Beschwerden – Kraft (MIMK)
DE Fuß: ROM (°)	13,6 ± 7,0	8,0 ± 2,3	*p* = 0,021*	R_s_ = −0,46; *p* = 0,03*	R_s_ = −0,21; *p* = 0,34
DE Fuß: MIMK (kgf)	0,1 ± 4,6	−2,3 ± 2,8	*p* = 0,301		

Die weitere Subgruppenanalyse zeigte, dass der ROM der DE im OSG in Fällen von Längenkorrekturen mit 11,2 ± 8,8 (4,9; 17,5) ° nicht signifikant kleiner war als in Fällen von reinen Achsen- oder Torsionskorrekturen 13,4 ± 4,4 (10,7; 16,0) °. Ebenso unterschieden sich die Differenz der MIMK zur jeweiligen geschlechterspezifischen 10 %-Normperzentile sowie die mittleren angegebenen Beschwerden (NRS) für die DE des Fußes nicht signifikant zwischen diesen Gruppen. In 13 der 23 nachuntersuchten Fälle zeigte sich am operierten Bein eine Muskelhernie, meist im Sinne einer langstreckigen, lediglich im Seitenvergleich auffallenden Prominenz der Extensorenloge. Hier zeigten ROM und MIMK keine signifikanten Unterschiede gegenüber Fällen ohne Muskelhernie. In der Gruppe mit Muskelhernie in der klinischen Nachuntersuchung waren die subjektiven Beschwerden der Fußhebung jedoch signifikant höher als in Fällen ohne postoperative Hernie (2,2 ± 1,0 vs. 1,3 ± 1,0, *p* < 0,05).

## Diskussion

Ziel der Studie war es, die Effekte der PF der Extensorenloge bei TO und Marknagelung hinsichtlich der subjektiven Funktion und der objektiven Parameter Kraft und ROM der DE im OSG in Fällen ohne präoperative Funktionseinschränkung zu untersuchen.

Durchschnittlich 6 Jahre postoperativ berichtete die überwiegende Mehrheit der Patient*innen über keine oder nur geringe subjektive Einschränkungen der Extensorenfunktion. Dass die Beeinträchtigung als am höchsten für die DE im OSG angegeben wurde, lässt sich vermutlich auch durch die höhere Alltagsrelevanz dieser Extensorenfunktion gegenüber den übrigen Extensoren erklären. Die wenigen Patient*innen, die eine mittlere oder hohe Beeinträchtigung der DE im OSG angaben, zeigten ein signifikant geringeres Ausmaß der DE (um 6–10°), verglichen mit Patient*innen mit geringerer subjektiver Beeinträchtigung. Folglich erscheint dieses Ausmaß der ROM-Minderung (bei einem Normalwert von etwa 25° DE) klinisch relevant, umso mehr als zwischen diesen Gruppen kein signifikanter Unterschied der MIMK festgestellt wurde.

Passend hierzu war keine signifikante Korrelation zwischen subjektiver Beeinträchtigung und MIMK festzustellen, und der Vergleich mit der geschlechterabhängigen 10 %-Normperzentile nach Huber et al. ergab im Falle festgestellter Kraftminderungen keine klinische Relevanz [8]. In Zusammenschau der Ergebnisse von Messungen und Befragungen halten die Autoren die Kraftminderung von 1,5 kgf (8,6 %) in der kleineren Stichprobe (*n* = 11) gegenüber der nichtoperierten Seite für klinisch nicht relevant. Die von Patient*innen berichtete Beeinträchtigung der DE nach TO mit PF ist am ehesten durch eine Abnahme der ROM im OSG bedingt, nicht durch eine Kraftminderung des M. tibialis anterior.

Die Subgruppenanalyse zeigte, dass weder die Art des Eingriffs (Achsen- und Torsionskorrektur vs. Längenkorrektur) noch die Entstehung einer anterioren Muskelhernie signifikanten Einfluss auf ROM und MIMK haben. Es ist davon auszugehen, dass diese Faktoren – einschließlich der kontinuierlichen Weichteildehnung über Wochen und Monate bei Kallusdistraktionen – weder per se funktionelle Probleme verursachen noch unsere vorherigen Ergebnisse beeinflussen.

Wir fanden zwar in Fällen mit postoperativer Muskelhernie eine signifikant höhere mittlere subjektive Beeinträchtigung gegenüber den Fällen ohne Muskelhernie, andererseits waren nicht signifikant mehr Fälle von Muskelhernien in der Gruppe von Patienten mit mittlerer und hoher Beeinträchtigung. Die Relevanz einer Muskelhernie für die subjektive Beeinträchtigung kann anhand unserer Ergebnisse nicht eindeutig geklärt werden. Bemerkenswert ist in diesem Zusammenhang, dass die nicht in die Studie eingeschlossenen Patient*innen mit Hypotrophien des Unterschenkels erfahrungsgemäß den geringen Volumengewinn des Unterschenkels begrüßen.

Ein direkter Vergleich der dargestellten Ergebnisse ist mangels vergleichbar aufgebauter Studien kaum möglich. Ergebnisse zur klinischen Funktion nach Marknagelung der Tibia ohne PF finden sich lediglich in Untersuchungen nach Tibiaschaftfrakturen: So fanden Obremskey et al. 12 Monate nach IM-Nagelung von Tibiaschaftfrakturen einen mittleren ROM der DE im OSG von 13,2°, ähnlich dem der vorliegenden Studie von 12,4° [14]. Lafaivre et al. beschreiben in ihren Langzeitergebnissen nach Marknagelosteosynthese eine ROM-Minderung im Seitenvergleich bei 14 von 33 Patient*innen, welche mehrheitlich weniger als 15° beträgt [12]. Unseres Wissens existieren bislang keine Studien zu Kraft und ROM nach TO zur Deformitätenkorrektur mittels Marknagel mit oder ohne PF.

Guillén-Rogel et al. beschreiben eine signifikante Korrelation zwischen Kraft und ROM der DE des Fußes (r_p_ = 0,47, *p* < 0,01) [7]. Die Korrelation zwischen der Differenz der Kraft der DE im OSG zur geschlechterspezifischen 10 %-Normperzentile und dem ROM war in unserem Kollektiv geringfügig stärker ausgeprägt (r_s_ = 0,58, *p* < 0,01).

Diese Studie ist nicht ohne Einschränkungen. So besteht bislang eine äußerst heterogene Studienlage zur Frage, ob intraindividuelle Kraftunterschiede auch in einer gesunden Bevölkerung aufgrund der Dominanz eines Beines vorkommen. Valderrabano et al. beschreiben eine signifikant höhere Kraft des dominanten Beines nur für die Plantarflexion [23]. Sepic et al. fanden eine signifikant höhere Kraft sowohl für die DE als auch für die Plantarflexion auf der Gegenseite der dominanten Hand bei Männern [16]. Moraux et al. wiederum beschreiben den rechten Fuß bei allen Patienten als stärker, mit signifikanten Ergebnissen für die Plantarflexion der Linkshänder und für die DE und Plantarflexion der Rechtshänder [13]. In weiteren Studien hingegen wurden gar keine signifikanten Kraftunterschiede zwischen dem rechten und dem linken Bein festgestellt [6, 7]. Die Dominanz des Beines wurde in unserer Studie nicht ausgewertet, aber dieser Faktor könnte die Ergebnisse des Kraftvergleichs mit der gesunden Seite beeinflusst haben.

Des Weiteren bezieht sich die durchgeführte Fallzahlplanung lediglich auf Kraftminderungen auf die 10 %-Normperzentile oder darunter. Für die Subgruppenanalyse kann somit nicht ausgeschlossen werden, dass die negativen Ergebnisse der Signifikanzprüfungen ganz oder teilweise auf die hier teils geringen Fallzahlen zurückzuführen sind.

Die zusammen mit der TO durchgeführte PF ist eine häufige operative Maßnahme in Verbindung mit Bohrlochosteotomien. Basierend auf den Ergebnissen dieser Studie sind mittelfristig nur sehr wenige Patient*innen von einer höhergradigen subjektiven Funktionsbeeinträchtigung betroffen, sodass aus Sicht der Autoren die Vorteile der PF mit TO zur Vermeidung eines postoperativen KS, dessen Folgen von einer dauerhaften Nervenschädigung (Spitzfuß) über Nekrosen und Kontrakturen bis zum Verlust von Gliedmaßen und Tode reichen können, die geringen Nachteile überwiegen.

Um einer dauerhaften Beeinträchtigung vorzubeugen, sollte die aktive und passive Beweglichkeit des OSG schon unmittelbar postoperativ beübt und in der Nachbehandlung regelmäßig überwacht werden. Zur Vervollständigung einer Risiko-Nutzen-Analyse wären Kohortenstudien mit großen Stichproben zur Verhinderung des postoperativen KS durch PF nötig.

## Fazit für die Praxis

- Bei der Mehrheit der Patient*innen nach Tibia-Osteotomien mit prophylaktischer Fasziotomie treten subjektive Einschränkungen im Alltag mittelfristig nicht oder nur gering ausgeprägt auf.
- Eine relevante subjektive Beeinträchtigung der Fußhebung ist am ehesten durch eine endgradige Bewegungseinschränkung bedingt, nicht durch eine Kraftminderung.
- Das Bewegungsausmaß sollte postoperativ regelmäßig geprüft und ggf. beübt werden.
- Der prophylaktischen Fasziotomie kommt angesichts der durch ein Kompartmentsyndrom drohenden dauerhaften Schädigungen ein hoher Stellenwert zu.

## References

[1] Bilen FE, Kocaoglu M, Eralp L, Balci HI (2010). Fixator-assisted nailing and consecutive lengthening over an intramedullary nail for the correction of tibial deformity. J Bone Joint Surg Br.

[2] Bohannon RW (1986). Test-retest reliability of hand-held dynamometry during a single session of strength assessment. Phys Ther.

[3] Bohannon RW (1999). Intertester reliability of hand-held dynamometry: a concise summary of published research. Percept Mot Skills.

[4] Bohannon RW, Andrews AW (1987). Interrater reliability of hand-held dynamometry. Phys Ther.

[5] Garfin SR, Tipton CM, Mubarak SJ, Woo SL, Hargens AR, Akeson WH (1981). Role of fascia in maintenance of muscle tension and pressure. J Appl Physiol.

[6] Geboers JF, van Tuijl J, Seelen HAM, Drost MR (2000). Effect of immobilization on ankle dorsiflexion strength. Scand J Rehabil Med.

[7] Guillén-Rogel P, San Emeterio C, Marín PJ (2017). Associations between ankle dorsiflexion range of motion and foot and ankle strength in young adults. J Phys Ther Sci.

[8] Huber E, Stoll T, Ehrat B, Stucki G (1997). Zuverlässigkeit und Normperzentilen einer neuen isometrischen Muskelkraftmessmethode. Physiotherapie.

[9] Janda V (2016). Manuelle Muskelfunktionsdiagnostik.

[10] Kenawey M, Krettek C, Liodakis E, Wiebking U, Hankemeier S (2010). Leg lengthening using intramedullay skeletal kinetic distractor: results of 57 consecutive applications. Injury.

[11] von Keudell AG, Weaver MJ, Appleton PT, Bae DS, Dyer GSM, Heng M, Jupiter JB, Vrahas MS (2015). Diagnosis and treatment of acute extremity compartment syndrome. Lancet.

[12] Lefaivre KA, Guy P, Chan H, Blachut PA (2008). Long-term follow-up of tibial shaft fractures treated with Intramedullary nailing. J Orthop Trauma.

[13] Moraux A, Canal A, Ollivier G, Ledoux I, Doppler V, Payan C, Hogrel J-Y (2013). Ankle dorsi-and plantar-flexion torques measured by dynamometry in healthy subjects from 5 to 80 years. BMC Musculoskelet Disord.

[14] Obremskey WT, Cutrera N, Kidd CM, Kidd CM (2017). A prospective multi-center study of intramedullary nailing vs casting of stable tibial shaft fractures. J Orthop Traumatol.

[15] Pagenstert G, Leumann A, Hintermann B, Valderrabano V (2008). Sports and recreation activity of varus and valgus ankle osteoarthritis before and after realignment surgery. Foot Ankle Int.

[16] Sepic SB, Murray MP, Mollinger LA, Spurr GB, Gardner GM (1986). Strength and range of motion in the ankle in two age groups of men and women. Am J Phys Med.

[17] Smith TO, Sexton D, Mitchell P, Hing CB (2011). Opening- or closing-wedged high tibial osteotomy: a meta-analysis of clinical and radiological outcomes. Knee.

[18] Spink MJ, Fotoohabadi MR, Menz HB (2010). Foot and ankle strength assessment using hand-held dynamometry: reliability and age-related differences. Gerontology.

[19] Stark T, Walker B, Phillips JK, Fejer R, Beck R (2011). Hand-held dynamometry correlation with the gold standard isokinetic dynamometry: a systematic review. PM R.

[20] Stoll T, Huber E, Seifert B, Michel BA, Stucki G (2000). Maximal isometric muscle strength: normative values and gender-specific relation to age. Clin Rheumatol.

[21] Thaller PH, Degen N, Fürmetz J, Wolf F (2017). Correction of length, alignment and torsion with fully implantable lengthening nails. Experiences with five different systems. Trauma Berufskrankh.

[22] Thaller PH, Fürmetz J, Degen N, Eilers T, Euler E, Wolf F (2019). Intraoperative customization of intramedullary nails—First results. Injury.

[23] Valderrabano V, Nigg BM, Hintermann B, Goepfert B, Dick W, Frank CB, Herzog W, von Tscharner V (2007). Muscular lower leg asymmetry in middle-aged people. Foot Ankle Int.

